# Modelling of Soft Connective Tissues to Investigate Female Pelvic Floor Dysfunctions

**DOI:** 10.1155/2018/9518076

**Published:** 2018-01-15

**Authors:** Aroj Bhattarai, Manfred Staat

**Affiliations:** Biomechanics Laboratory, Institute of Bioengineering, Aachen University of Applied Sciences, 52428 Jülich, Germany

## Abstract

After menopause, decreased levels of estrogen and progesterone remodel the collagen of the soft tissues thereby reducing their stiffness. Stress urinary incontinence is associated with involuntary urine leakage due to pathological movement of the pelvic organs resulting from lax suspension system, fasciae, and ligaments. This study compares the changes in the orientation and position of the female pelvic organs due to weakened fasciae, ligaments, and their combined laxity. A mixture theory weighted by respective volume fraction of elastin-collagen fibre compound (5%), adipose tissue (85%), and smooth muscle (5%) is adopted to characterize the mechanical behaviour of the fascia. The load carrying response (other than the functional response to the pelvic organs) of each fascia component, pelvic organs, muscles, and ligaments are assumed to be isotropic, hyperelastic, and incompressible. Finite element simulations are conducted during Valsalva manoeuvre with weakened tissues modelled by reduced tissue stiffness. A significant dislocation of the urethrovesical junction is observed due to weakness of the fascia (13.89 mm) compared to the ligaments (5.47 mm). The dynamics of the pelvic floor observed in this study during Valsalva manoeuvre is associated with urethral-bladder hypermobility, greater levator plate angulation, and positive Q-tip test which are observed in incontinent females.

## 1. Introduction

The structure of the female pelvic floor is an interrelated system of bony pelvis, muscles, fasciae, and ligaments with multiple functions. Mechanically, the pelvic organ support system is of two types:* supporting* system of the levator ani (LA) muscle and the* suspension* system of the endopelvic fascia [1, 2]. At normal resting conditions, the soft and intact connective tissue holds the pelvic organs to store urine and stool which relaxes in demand to allow the organ mobility for excretion. On the other hand, the muscle system provides normal resting contraction and reflex contraction to maintain the urogenital hiatus [3].

During pregnancy, the pelvic muscles and the connective tissues undergo softening induced by hormonal changes which widens the way out for fetal descend during vaginal delivery [4, 5]. Muscles normally regain shape and function within a range of few days to months [6, 7]. However, significant denervation injury of the pelvic floor musculature during childbirth stresses the soft vaginal hammock and the ligaments to support the pelvic organs [8]. In addition, with the onset of menopause, estrogen and progesterone hormones are progressively reduced which are protective against tissue deterioration and are important to maintain the tissue integrity. Collagenase activity or collagen metabolism triggered by a family of zinc-dependent endopeptidases known as the matrix metalloproteinases (MMPs) in the extracellular matrix alters the collagen ratios thereby decreasing the stronger and thicker collagen type I relative to weaker and distensile collagen type III [9]. Furthermore, active remodelling of the tissues also modifies the smooth muscle cells thereby reducing their content and architecture [10]. This modification is profoundly associated with laxity of the tissue due to loosely arranged collagen fibres, less dense extracellular matrix, and impaired smooth muscle cells.

The loose insertions of the striated pelvic muscles due to the depolymerization of the collagen fibrils of the vaginal hammock tissues fail to transmit the muscle contraction to the organs and implications are seen in the form of pelvic floor dysfunction (PFD) [11]. Pelvic organ prolapse, incontinence, and sexual dysfunction are some of the dysfunctions which progress with menopause and age [12]. Among them, stress urinary incontinence (SUI) is an artefact situation in aging female where significant urine leakage occurs involuntarily during sudden increase in intra-abdominal pressure. The cause for SUI in old ages is weak support at the mid-urethra and failed bladder neck closure from the collagen deficient pubourethral ligament, vaginal hammock, vaginal wall, and denervated or damaged levator ani [1, 13].

About 20–40% of the world female population are estimated to be affected by stress urinary incontinence [14]. Surveys (in France, Germany, and UK) showed that 41% of the American and more than 30% of the European female population are affected by SUI [15]. Unfortunately, the prevalence of urinary incontinence (UI) is difficult to determine; many women do not seek medical advice and treatment because of embarrassment and misconception regarding treatment [16, 17].

For more than a century, the anatomy and relative contribution of the endopelvic fascia to the support system and dysfunctions have been a subject of research and controversy. Several computational models have appeared to study the phenomenon of the pelvic floor dysfunctions focusing mainly on the anatomy of the pelvic viscera, dense fibromuscular ligaments, and the muscles [18–23]. Since it is difficult to identify the thin and continuous pelvic fascia unit via available computer techniques, these computational studies have adopted simplified geometries and mathematical modelling. Also, wherever studied as an integrated structure, alteration of the fascia constituents, mainly elastin-collagen fibres, adipose tissue and smooth muscles could greatly influence the overall tissue mechanics. Therefore, as a next step, we have considered the soft tissue mechanics at microscopic level using the mixture model.

On this basis, this study describes improvements in a 3D finite element model of the female pelvic floor which considers the realistic support of the organs at the pelvic side walls, employs the improvement of our previous FE model [24, 25], and incorporates the realistic anatomy and boundary conditions of the endopelvic (pubocervical and rectovaginal) fascia. In the mechanical part, this paper considers the mechanics of major tissue constituents to characterize the mechanical behaviour of the fascia using a constitutive mixture model which is weighted by the volume fractions of the components and studies the influence of varying constituents, focusing mainly on the stiffest collagen to model the tissue impairment/weakness/laxity and different classes of the endopelvic fascia in craniocaudal direction [26, 27]. Several computations are carried out with the presented computational model with healthy and damaged supporting tissues, and comparisons are made to understand the pathophysiology of the SUI.

## 2. Sheet Plastination to Finite Element Models

### 2.1. NURBS-Based Geometry

**Figure 1:**
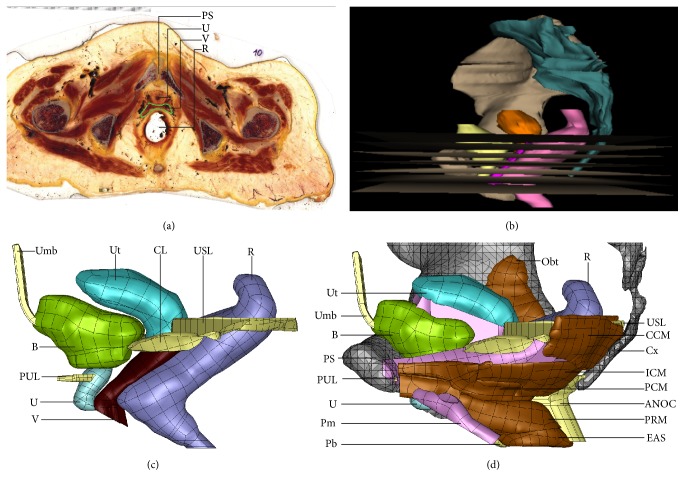
(a) Epoxy (E12) slice of the female pelvis (provided by Feil and Sora, Vienna) [29] and (b) three-dimensional visualization model reconstructed using WinSurf software (3D tool) with horizontal gray plastination slices at different heights. Smoothed female pelvic NURBS geometries of the organs suspended by (c) ligaments (yellow), (d) fasciae (pink), and (c) muscles (brown) in sitting position. The rigid pelvic bone is included here only to show the attachment of the pelvic muscles, fasciae, and ligaments.

A three-dimensional biomechanical model of the female pelvic floor was reconstructed from a 70-year-old female cadaver specimen with no known history of pelvic pathology. The detailed methodology of creating the computer model of the internal morphology from the plastinated slices has been described in [24, 28, 29]. Using the ultra-thin E12 sheet plastination technique (Figure 1(a)), the visualization model (Figure 1(b)) is well suited to map the pelvic floor anatomy into a finite element model for biomechanical analysis. Due to the complex geometry, reconstructed triangular surfaces show artefacts such as high aspect ratio, holes, and intersecting faces. Finite element (FE) simulations are known to fail or at least affect the convergence due to distortion of the mesh under extreme deformations. The convergence problems of bad shaped finite elements can be overcome by the newly proposed smoothed finite element method which has been applied also to problems of soft tissues [30, 31]. Nonsmooth surface of the geometric model of the organ is the most relevant problem because it will prevent convergence in the analysis of the contacts between organs and self-contacts of hollow organs. Preliminary smoothing of the triangular FE mesh is done by using the 3D mesh processing software MeshLab (http://meshlab.sourceforge.net). The Rhino software (https://www.rhino3d.com) is used to repair and transform the irregular surfaces into smooth free-form surfaces based on nonuniform rational B-splines (NURBS) as shown in Figures 1(c) and 1(d). NURBS are much easier to handle and provide smooth geometrical models for the FE simulation. The smoothed geometries are then imported into the open source preprocessor and postprocessor software Salome (http://www.salome-platform.org) to create FE meshes.  Table 1 lists the abbreviations used in the figures.

### 2.2. Endopelvic Fascia as a Single Unit

**Figure 2:**
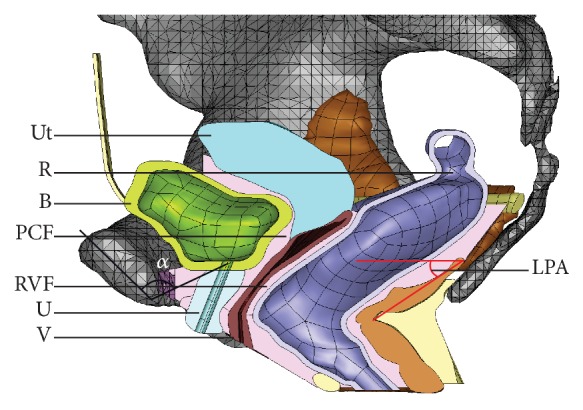
The organs, fasciae (pink), and muscles are cut in the sagittal plane. For comparison of the fascia geometry refer to [26]. The angle *α* measures the angle between the midpubic line to the bladder neck. Levator plate angle (LPA) is an angle subtended by the initial take-off portion of the iliococcygeus from the coccyx to a horizontal reference line. PCF: pubocervical fascia and RVF: rectovaginal fascia.

It must be emphasized that the endopelvic fascia is a* single unit* of loose network of connective tissue strand. It plays an important role to provide a firm base to maintain the proper position of the urethrovesical junction, particularly during straining, to fill the organ-organ and the muscle-organ spaces in the pelvic floor by suspending the organs to the pelvic wall and muscles [26], and to prevent the urethral and the bladder hypermobility [32]. Several regions of the endopelvic fascia and its associated peritoneum have been named with respect to their support at the adjacent structures. They are pubocervical fascia, rectovaginal fascia, Waldeyer fascia, rectosacral fascia, mesorectum, paracolpium, pubourethral ligament, cardinal ligament, uterosacral ligament, and umbilical ligament. The listed ligaments are the real condensations/thickening of the endopelvic fascia rather than true ligaments [33]. Since geometrical construction of such thin endopelvic fascia by conventional radiological techniques is not possible yet, most of the computational studies [19–21] present ligaments as supporting structures of the organs without considering the thickening of the connective tissue network. For a more realistic mechanical structure, this study models fasciae as continuous tissue network and the respective thickening at different anatomical regions to the ligaments (Figure 2).

The endopelvic fascia is a heterogeneous network of collagen, elastin, nerves, lymph channels, and nonvascular smooth muscle fibres [34] extending from the pubic symphysis to the sacrum and the ischial spine. With respect to the vaginal support, DeLancey described the endopelvic fascia in three different anatomical levels [35]. Level I refers to the intermingling fibres of the cardinal/uterosacral ligament complex which attaches the upper vagina, cervix, and lower uterine segment to the obturator muscle/sacrum, piriformis, and coccygeus, respectively (Figure 2). Laxity in this complex may result in abnormal bladder emptying and uterine prolapse [36]. Level II supports the middle one-half of the vagina to the levator ani muscles and provides a firm base for bladder neck and urethra. Loss of support at this level results in cystourethrocele, urethral-bladder hypermobility, and SUI. Level III supports the distal vagina to maintain the anatomic positions of the vagina and urethra by fusion with the perineal structures (Figure 2).

### 2.3. Pelvic Fascia Constituents

**Figure 3:**
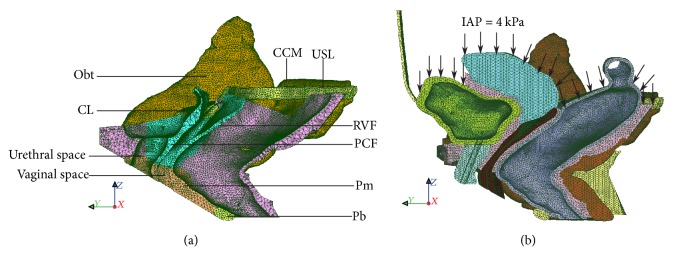
Sagittal section of the 3D finite element mesh showing (a) pubocervical fascia (PCF) and rectovaginal fascia (RVF), ligaments and perineal structures, and (b) FE mesh loaded with intra-abdominal pressure (IAP). Endopelvic fascia is separated with different amount of elastin and collagen constituents, blue (elastin rich) and pink (collagen rich).

Histological studies found lax urethral support due to reduced collagen content on the SUI patients which did not appear on the cystocele subjects [32, 37]. Petros [27] suggested that the distal 2-3 cm of the endopelvic fascia around the vaginal length is collagen rich, and the superior part is rich in elastin and smooth muscle. This lights up the concept of the phenomenal differences of the urinary incontinence and (bladder and urethral) prolapse. Laxity of the PUL and the collagenous urethral support fascia fails to close the urethra during straining. Funneling of the proximal urethral occurs to leak the urine due to active longitudinal anal muscle contraction, whereas laxity or paravaginal damage in the upper elastic fascia drags the bladder into the vaginal canal commonly known as cystocele. The presented model, thereby, adopts two different regions of the pubocervical and rectovaginal fascia with varying tissue compositions to describe different PF disorders mechanically (Figure 3(a)).

### 2.4. Finite Element Mesh of the Female Pelvic Floor

Geometries of every pelvic structure are assembled and volume discretized to create three-dimensional FE meshes considering the significant thickness of each pelvic constituent. A smooth FE mesh as shown in Figure 3 is constructed from 65,6563 linear tetrahedrons (4-node elements) except for the vagina, which has been discretized with 28,606 quadratic tetrahedral (10 nodes) elements for high smoothness in the analysis of self-contact between the anterior and posterior vaginal walls. The integrated FE model of the female pelvic floor consists of 24 structures: 8 ligaments, 5 organs, 8 muscles, 1 fascia, and 2 perineal structures (perineal membrane and perineal body) (Figure 3).

### 2.5. Boundary Conditions

The endopelvic fascia is connected to the pelvic diaphragm providing a firm support to the organs.* Pelvic bones are rigid structures stiffer than pelvic tissues by orders of magnitude*. Therefore, they are included in the model as rigid points for the fixation of the muscles, ligaments, and fasciae. The superior end of the umbilical ligament is connected to the umbilicus and is fixed for any movement. The piriformis muscle does not support the IAP and is not included in the model. However, the superior surfaces of the coccygeus muscles are connected to the piriformis and constrained in the horizontal plane. A frictionless sliding contact between the two walls of the vagina is considered.

The IAP of 40 cm of H_2_O and 100 cm of H_2_O during supine Valsalva manoeuvre and straining manoeuvre are applied on the upper surface of the organs [38]. During normal micturition, the urethra is subjected to an average fluid pressure of 60 cm of H_2_O to open the bladder neck [39]. Considering the linear anatomic profile of the urethra, the fluid pressure in the finite element simulation is dropping linearly from 60 cm of H_2_O = 0.0058 MPa at the bladder neck to zero at the urethral exit. The inner surface of the bladder is also subjected to the same fluid pressure in the simulation. Based on the* Integral Theory*, the lateral vaginal wall transmits muscle contractions against suspensory ligaments; levator plate (LP) pulls the vagina and the bladder neck posteriorly against the PUL fulcrum, and the longitudinal muscle of anus (LMA) pulls downward against the USL. Ligaments and fasciae with laxity or weakness or reduced stiffness fail to maintain the normal position of the organs, which results in incontinence and prolapse.

### 2.6. Soft Tissue Mechanics

Pelvic connective tissues are made up of cells linked by the extracellular matrix (ECM). While the cells provide biochemical functions, the noncellular ECM provides (structural and mechanical) support and physical scaffolding for the cellular constituents. It is mainly composed of macromolecules of biopolymers including elastin-collagen fibres and a varying amount of adipose (fat) tissue and smooth muscle organized in a nonhomogeneous fashion to form a complex composite microstructure. At structural level, its biomechanical behaviour depends on the components, since each of them possesses unique mechanics to one another; the collagen significantly contributes to the stiffening response (limited extension or stretch), elastin to the distensibility, and the smooth muscle to the continuous contractility, and the softest adipose tissue serves as a cushion with much lower stiffness than the other constituents. In this section, an attempt is made to describe the* theory of immiscible mixture* where the tissue constituents retain their unique identity and mechanics but provides an integrated effect to the structural tissue mechanics. The computational model assumes that the composite materials can be represented by the periodic repetition of a microstructure, usually known as a representative volume element (RVE). Using a homogenization approach, the overall mechanical response of any nonlinear multiconstituent soft tissues can be estimated.

#### 2.6.1. Assumptions on the Mathematical Modelling of Fascia

  The fibres contributing to the mechanical strength are curled in a 2D plane of the loading axis.
  The contribution of the smooth muscle (around 3% in strongest ATFP ligaments [9]) to load bearing is passive and negligible.
  Every constituent of the fascia is assumed isotropic, nonlinear elastic, and incompressible.
  There are no frictional interactions between the components to avoid energy loss during the deformation process.
  Constituents satisfy the classical balance relations.

### 2.7. Model Formation

#### 2.7.1. Single Hyperelastic Phase

Macroscopically, the highly nonlinear, incompressible, and elastic stress-strain relation of every soft biological tissue can be derived using the* Helmholtz free-energy function *(*W*) defined per unit reference volume. The scalar-valued energy function is referred to as the strain energy function or stored energy function if it depends only on the deformation gradient **F**; that is, *W*≔*W*(**F**) [40]. For incompressible hyperelastic materials, the strain energy function can be postulated as

(1) $$\begin{array}{c} W=W\left(\;F\;\right)-p\left(\;J-1\;\right), \end{array}$$

where the scalar *p* is an indeterminate* Lagrange multiplier* identified as a hydrostatic pressure and can be determined from the equilibrium equations and the boundary conditions. The Jacobian determinant *J* = det⁡[**F**] measures the ratio between the deformed volume *V* and the undeformed volume *V*_0_; that is, *J* = *V*/*V*_0_. The derivative of the scalar-valued function *W* with respect to the tensor variable **F** gives a second-order gradient tensor also known as the first Piola-Kirchhoff stress tensor **P**:

(2) $$\begin{array}{c} P≔\frac{\partial W\left(F\right)}{\partial F}-\frac{\partial p\left(J-1\right)}{\partial C}. \end{array}$$

In reference or stress-free configuration, the deformation gradient equals the identity tensor rendering the strain energy function zero; that is,

(3) $$\begin{array}{c} W=\left\{\begin{array}{cc} W\left(I\right)=0, & \text{if}\;\;F=I\\ \geq 0, & \text{if}\;\;F\neq I. \end{array}\right. \end{array}$$

The deformation gradient tensor can be decomposed into positive definite and symmetric right material stretch (**U**) or left spatial stretch tensor (**V**) and a rotation tensor (**R**) as **F** = **RU** = **VR**. Here, **R** is proper orthogonal satisfying **R**^*T*^**R** = **I** and det⁡[**R**] = 1. Introducing the symmetric and positive definite right Cauchy-Green stretch tensor **C** = **F**^*T*^**F** = **U**^2^, the strain energy function can be alternatively expressed in terms of **C** (for simplicity of notation the name of the function *W* is not changed)

(4) $$\begin{array}{c} W≔W\left(\;F\;\right)\equiv W\left(\;C\;\right). \end{array}$$

Similarly, according to the Doyle-Ericksen formula, the strain energy function differentiated with respect to the Cauchy-Green stretch tensor gives the second Piola-Kirchhoff stress tensor

(5) $$\begin{array}{c} S=2\frac{\partial W\left(C\right)}{\partial C}-pC^{-1}. \end{array}$$

For isotropic hyperelastic materials, the deformation gradient in uniaxial tension **F** is given as

(6) $$\begin{array}{c} F≔\left[\begin{array}{ccc} \lambda_{1} & 0 & 0\\ 0 & \lambda_{2} & 0\\ 0 & 0 & \lambda_{3} \end{array}\right], \end{array}$$

where the scalar values *λ*_*i*_ are the stretch ratios in three-coordinate axis. The strain energy function can be expressed in terms of the principal invariants (*I*_1_, *I*_2_, *I*_3_) of the stretch tensor (**C**), such that

(7) $$\begin{array}{c} W\left(\;C\;\right)=W\left(\;I_{1}\left(\;C\;\right),I_{2}\left(\;C\;\right)\;\right)-p\left(\;J-1\;\right), \end{array}$$

with *I*_1_(**C**) = tr⁡[**C**], *I*_2_(**C**) = (1/2){(tr⁡[**C**])^2^ − tr⁡[**C**^2^]}, and *I*_3_(**C**) = det⁡[**C**] = det⁡[**F**] = *J*^2^. In terms of the principal stretches, the invariants are in the form

(8) I1C=λ12+λ22+λ32,I2C=λ12λ22+λ22λ32+λ32λ12.

The second Piola-Kirchhoff stress in terms of invariants can be derived using chain rule as

(9) S=2∂WC∂C−pF−1F−T=2∑i=1,2,3∂W∂IiC∂IiC∂C−pC−1.

The derivatives of the invariants with respect to **C** are

(10) ∂I1∂C=I,∂I2∂C=I1I−C.

Substituting the partial derivatives in (9), the constitutive model to characterize the isotropic hyperelastic materials at finite strain can be given as

(11) S=2∑i=1,2∂W∂Ii∂Ii∂C−pC−1=2∂W∂I1+I1∂W∂I2I−∂W∂I2C−pC−1.

In the current or spatial configuration, the true or Cauchy stress *σ* uses the left Cauchy-Green strain tensor. It follows the second Piola-Kirchhoff stress **S** by the Piola transformation *σ* = *J*^−1^**FSF**^*T*^ to get

(12) $$\begin{array}{c} \sigma =2J^{-1}\left[\;\left(\;\frac{\partial W}{\partial I_{1}}+I_{1}\frac{\partial W}{\partial I_{2}}\;\right)B-\frac{\partial W}{\partial I_{2}}B^{2}\;\right]-pI, \end{array}$$

where **B** = **FF**^*T*^ = **V**^2^ is the left Cauchy-Green stretch tensor.

#### 2.7.2. Mixture of Hyperelastic Phases

Continuum modelling of the biological tissues is notoriously intimidating as it possesses a number of challenges related to its structure and composition. Modelling the overall mechanical response of any multiple constituent material, such as connective tissue, needs to obey individual balance and constitutive relations by each constituent and to agree for mass, momentum, and energy by the overall mixture [41]. In general terms, the mixture as a whole should behave as a pure substance with an exchange of mass, momentum, and energy between constituents. In addition, based on the continuum theories of heterogeneous substances, all constituents coexist at every point in the microstructurally small volume also known as representative volume element (RVE). Soft tissue RVE is composed of elastin-collagen fibres, fat tissues, and smooth muscles which are weighted by their volume fractions (*f*_*i*_). For the homogenization of such heterogeneous biological materials and to determine the effective mechanical parameters, classical Voigt upper bounds and Reuss lower bounds have been widely used. The Voigt isostrain mixture rule has some technical advantage over the Reuss isostress rule [42–45]. The SEF of the soft tissue (*W*) is the sum of the SEF of the constituents; that is,

(13) $$\begin{array}{c} W=\underset{i=fas,ad,sm}{\sum }\left(\;f_{i}W_{i}\left(\;C\;\right)-p\left(\;J-1\;\right)\;\right). \end{array}$$

In Voigt's isostrain rule, the deformation of the constituents is kinematically compatible; that is, they have the same **C** so that

(14) WVoigt=∑i=fas,ad,smfiWiC−pJ−1=ffasWfasC+fadWadC+fsmWsmC−pJ−1,

where *f*_fas_, *f*_ad_, and *f*_sm_ are the volume fractions of the elastin-collagen fascia, adipose tissue, and smooth muscle satisfying the total volume fraction *f* = *f*_fas_ + *f*_ad_ + *f*_sm_ = 1. Authors would like to emphasize that the strain energy for the smooth muscle is solely because of the passive contribution, no activation of the muscle considered, so that (*σ*_active_)_sm_ = 0. Soft tissues and their constituents can be considered as incompressible due to abundance of water. For such materials, *J* = 1 so that the SEF expression (7) depends only on the first two invariants and an additional workless reaction as

(15) $$\begin{array}{c} W\left(\;C\;\right)=W\left(\;I_{1}\left(\;C\;\right),I_{2}\left(\;C\;\right)\;\right)-p\left(\;J-1\;\right). \end{array}$$

Several hyperelastic material models are available, for example, Neo-Hookean, Arruda-Boyce, Mooney-Rivlin, and Ogden models. We present here the simplest models (the Mooney-Rivlin type polynomial function) which have been typically applied for many biological tissues. Elastin and collagen fibres are sparsely distributed in the matrix of the soft tissues, and several anisotropic constitutive models have been implemented to characterize their mechanical behaviour [44, 46]. Intense histological study is required to evaluate the proportion, orientation, and distribution of the tissue constituents for soft fascia and is yet to be done. Therefore, in this study, the elastin-collagen fascia, adipose, and smooth muscle are assumed to be isotropic, hyperelastic, and incompressible materials and are modelled with a three-term polynomial strain energy function* i* = fas, ad, sm:

(16) $$\begin{array}{c} W_{i}=C_{10}^{i}\left(\;I_{1}-3\;\right)+C_{01}^{i}\left(\;I_{2}-3\;\right)+C_{20}^{i}{\left(\;I_{1}-3\;\right)}^{2}. \end{array}$$

The total analytical 2nd Piola-Kirchhoff stress tensor **S** is derived at the tissue or macroscopic level by using differential equations (9) and (10) in the initial configuration which is written as

(17) S=ffasSfas+fadSad+fsmSsm−∂pJ−1∂C=2∑i=fas,ad,sm  ∑j=1,2fi∂Wi∂Ij∂Ij∂C−pF−1F−T=2∑i=fas,ad,smfi∂Wi∂I1+I1∂Wi∂I2I−∂Wi∂I2C−pC−1.

For an incompressible hyperelastic material with preserved volume (*λ*_1_*λ*_2_*λ*_3_ = 1) under uniaxial tension, *λ*_1_ = *λ* gives $\lambda_{2}=\lambda_{3}=1/\sqrt{\lambda }$. Then the isochoric or distortional first Piola-Kirchhoff or nominal stress **P** along the stretch direction can be written as

(18) P=∑i=fas,ad,smfi2C10iλ−1λ2+2C01i1−1λ3+4C20iλ−1λ2λ2+2λ−3.

**Figure 4:**
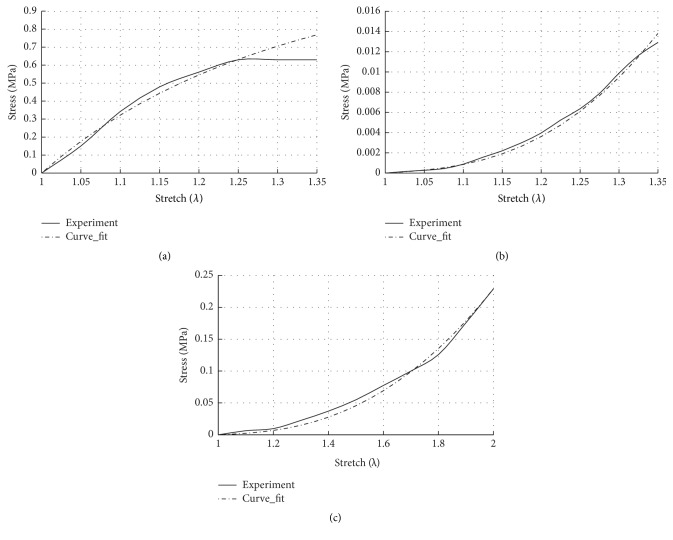
Fitting of the nominal stress (*P*) versus stretch (*λ*) curve for (a) collagen-elastin fascia [47], (b) adipose tissue [50], and (c) smooth muscle without active contractility is assumed during micturition [39] using three-term polynomial strain energy function (16).

The constants *C*_10_^*i*^, *C*_01_^*i*^, *C*_20_^*i*^ of the elastin-collagen fascia, adipose, and smooth muscle are fitted from the tensile experiments adopted from literature and are listed in Table 2.  Figure 4 shows the capability of the three-term hyperelastic polynomial function to capture the mechanical response of experimented tissue components. The fascia is a very thin structure measuring from 1.43 ± 0.41 mm to 1.56 ± 0.17 mm [47]. This makes it difficult to visualize it in medical imaging and to represent it in an FEM model. It is combined with surrounding tissues to a thicker “organ” which is modelled as a composite material in (18). It is not easy to determine the exact proportion of the tissue constituents. Separate staining protocols are necessary for each component and histological studies are not consistent. For example, for structurally similar uterosacral and ATFP ligaments, wide ranges of collagen densities (23% [48]–48.75% [49] versus 84% [9]) and smooth muscle cells (20% [48] versus 5% [9]) were measured. At this stage, with the personal advice* from the experience of anatomist and urologist Professor Dr. med. Mircea Constantin Sora* (Sigmund Freud University Vienna, Austria) approximation of the fascial components was done to have a more realistic model. In a total volume of the endopelvic fascia, abundance of the adipose tissue is assumed (≈85%) and serves as a cushion to the adjoining organs. The thin layer of the fascia formed by elastin and collagen fibres embedded in the ground matrix occupies about 10% of the endopelvic fascia volume, whereas the remaining 5% is estimated to be occupied by the randomly distributed smooth muscles. Therefore, the mechanical behaviour of the fat-free endopelvic fascia itself consisting of elastin and collagen embedded in the ground matrix is adopted from the curve fit of the uniaxial tensile experiment performed on the human transversalis fascia [47] which is continuous with the pelvic fasciae. For adipose tissue and smooth muscle, corresponding literature [39, 50] was reviewed and fitted with (18). Finally, the stress-stretch relationship of the composite fascia tissue, made up of elastin-collagen fascia, adipose tissue, and smooth muscle is given by (18) and its behaviour can be defined by fitted three hyperelastic parameters *C*_10_, *C*_01_, *C*_20_.

### 2.8. Mechanical Modelling of Pelvic Floor Tissues

*The stress-strain curves of all pelvic structures are adopted from experiments published in literature and are fitted with three-term polynomial functions *(15)* to obtain isotropic hyperelastic parameters which are later used for the numerical study *(Table 3)*. Literally, it is not easy to obtain a stress-strain relation for all tissues which consider anisotropy, wherever possible, as it is rather difficult to cope up with the FE software because all anisotropic nonlinear mechanical characterization is not readily available *[51, 52]. Therefore, focus is given on the simple isotropic but widely adopted nonlinear hyperelastic material behaviour. The parameterization of the mechanical behaviour of the endopelvic fascia with different constituents has not been found yet. In order to estimate the mechanical behaviour of elastin-rich fascia and collagen-rich fascia along the vaginal length, comparison has been made from experiments after digesting or removing either collagen or elastin from artery, elastin-rich spleen, and collagen-rich liver [53–55]. Tissue stiffness is found to be substantially varying after digestion. If elastin is removed, the initial stiffness at the toe region is greatly reduced with an immediate linear region due to the recruitment and alignment of the leftover collagen fibres. In such remodelled tissues, the specimen distensibility (only 4%) comes only from the rearrangement of the inelastic collagen fibres. Similarly, after collagen reduction samples show nearly linear stress-stretch curves with very low overall stiffness and are highly stretchable up to 70% due to elastin fibres. Though no analytical relation has been discussed in both publications to describe the relative changes in the mechanical response from elastin-collagen reduction, comparing the experimented stress-stretch curves of the collagen digested tissue showed stiffness reduction by more than 90% compared to the elastin digested tissue.

In the healthy endopelvic fascia, elastin and collagen are only varying along the vaginal wall. Other tissue components (adipose and smooth muscle) can be assumed to be unchanged. Thus, 90% or more stiffness reduction due to complete removal of other components would be an overestimation for the fascia composition. Therefore, we adopted an average stiffness difference of 75% between the elastin and the collagen-rich regions of the endopelvic fascia, thus maintaining the general nonlinearity of the stress-stretch curve. In addition, the microstructure of the smooth muscle and adipose is homogeneous. These components can be assumed to be entirely elastic in passive stretching and their contribution in the mechanical response of the endopelvic fascia can be estimated from their respective stress-stretch curves (Figure 4).  Section 2.9 provides more information about the influence of collagen in soft tissues.

Though soft tissues are composed of elastin and collagen fibres, anisotropy associated with such tissues is the main difficulty in the biomechanical modelling. Multiaxial deformation tests have not been performed for all pelvic tissues; only few specimens extracted either from humans or from animals are tested in such way [56–60]. Assuming the pelvic structures are dominantly subjected to uniaxial tension during physiological pelvic movement and the required function in this study is the load carrying and not the function of the organ themselves, an isotropic formulation and the curve fit of the pelvic structures (Table 3) adopted in this study can provide realistic representation of the load transfer in the pelvic floor. For comparison readers may refer to cited literature in Table 3.

### 2.9. Reduced Tissue Stiffness due to Collagen Degradation

Collagen is the most abundant protein in the human body and is an important structural unit of soft tissues. Among 20 different types, types I and III are substantial in soft tissues which together copolymerize to form fibrils with controlled diameters, which influences the biomechanical characteristics of a given tissue when stressed [65, 66]. Type I collagen provides mechanical strength to tissues while type III contributes to elasticity and regulates collagen fibril diameter during fibrillogenesis. With the onset of menopause, estrogen and progesterone hormones are progressively reduced which are protective against tissue deterioration and important to maintain the tissue integrity. A family of zinc-dependent endopeptidases known as the matrix metalloproteinases (MMPs), especially active MMP13, then triggers the collagenase activity. Such collagenase activity or collagen metabolism in the extracellular matrix alters the collagen ratios thereby decreasing the stronger and thicker collagen type I relative to weaker and distensile collagen type III [9]. Reduced ratio of collagen types I to III leads to thinner collagen fibrils, pathologically altered extracellular matrix (ECM), abnormal quantity and organization/alignment of collagen fibres, and increase of the nonpolymeric soluble collagen. Significant reduction of (68%) total collagen in pelvic floor disorder subjects has been observed [49].

Furthermore, active remodelling of the tissues also modifies the smooth muscle cell [10] thereby reducing and disorganizing the smooth muscle content. In contrast, no strict evidence about significant elastin metabolism and reduction of the elastin content and fibre size is observed during tissue metabolism [67]. Thus, the change of the biomechanical behaviour of the tissue observed as laxity or reduced stiffness is profoundly associated with loosely arranged collagen fibres, less dense extracellular matrix, and impaired smooth muscle cells [48].

To simulate the progressive development of the PFDs, ligaments and fasciae are successively impaired/weakened between 0% and 95%. The tissue impairment is modelled as reduced material stiffness obtained by multiplying the respective material parameters in Table 3 from 0.9 (10% impairment), 0.8 (20% impairment), and 0.7 (30% impairment) up to 0.05 (95% impairment). Similar methodology of tissue impairment has been adopted by Brandão et al. [22], Chen et al. [19], and Luo et al. [20].

## 3. Results and Discussion

In this section, we present some results of the computer simulations which test the capabilities of the FE model. The finite deformation simulations for the computational model (Figure 3) are performed with the open source FE software,* Code_Aster* (https://www.code-aster.org). The characteristic displaced positions of the pelvic organs are compared with their initial position at rest and verified with medical images (Figure 6) and clinical measurements. The study parameters are the vertical (VUVJ) and horizontal (HUVJ) urethrovesical junction $\left(UVJ=\sqrt{{\left(VUVJ\right)}^{2}+{\left(HUVJ\right)}^{2}}\right)$ movements, the urethral axis with the vertical (Ur), and the angle alpha *α* between the midpubic line and the bladder neck. The stiffness of the supporting endopelvic fascia and ligaments is sequentially reduced between 0% and 95% to simulate possible pelvic floor disorders due to tissue laxity or weakening. The computational results presented here show the relative movement of the pelvic organs due to combined effects of the IAP, the detrusor pressure, impairment of the supporting system, and the active muscle opening forces as suggested by Petros in his so-called* “Integral Theory.”* The results presented in this study offer the capability of a robust computational model of the female pelvic floor which poses a good start to improve our understanding of the SUI. The findings presented in this study can be used to validate against medical examinations and surgical interventions that can profoundly improve both the health and quality of life of aging women.

### 3.1. Mobility of the Urethrovesical Junction

**Figure 5:**
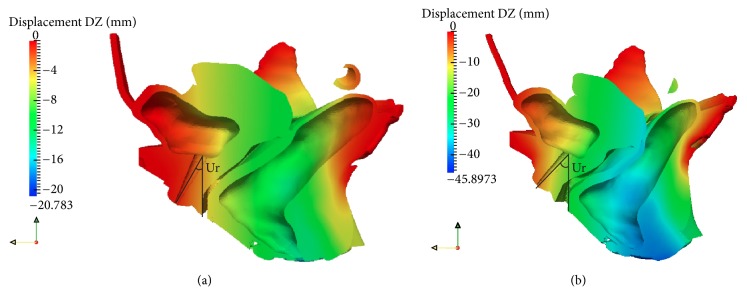
Sagittal section of a 3D pelvic model with results of the FE simulation showing the vertical movement of the pelvic floor during Valsalva manoeuvre for (a) healthy tissues and (b) 95% weakened ligaments and fasciae. Urethral axis (Ur) > 30° with vertical line resembles positive Q-tip test. A great vertical descent (DZ = 38.79 mm) of the anal orifice is observed in a dynamic MRI in a SUI patient [76]. The vertical displacement of the anal orifice in the SUI calculation (Figure 6(b)) is 40.83 mm.

**Figure 6:**
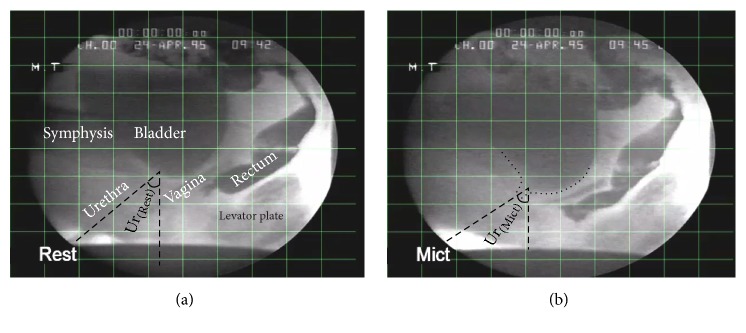
X-ray examination of an asymptomatic female pelvic floor urethral angle (a) Ur_(Rest)_ at rest and (b) Ur_(Mict)_ during micturition [77].

**Figure 7:**
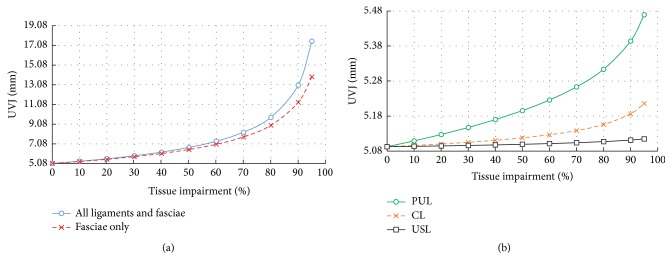
Effects of tissue impairment* (reduced stiffness)* on the vertical movement of the UVJ for the impairment of (a) all connective tissues (fasciae and ligaments) (b) and caused by individually impaired ligaments.* Maximum organ dislocation is achieved due to weakness in fasciae rather than the ligaments*.

**Figure 8:**
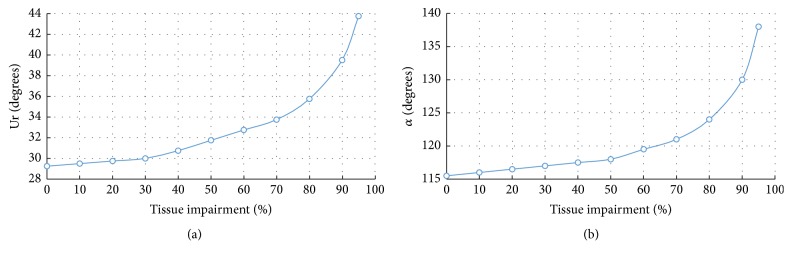
Plot of increased fascia impairment* (reduced stiffness)* on (a) urethral axis, Ur, and (b) movement of the UVJ, (*α*).

The impact of weakened pelvic support system during strain on the movement of the urethrovesical junction in women with SUI is illustrated in Figures 5–8. For all the simulation cases, the resting position of the urethrovesical junction (*α*_Rest_ = 110° in Figure 2) and the urethral angle (Ur_(Rest)_ = 22° in Figure 6(a)) included between the urethral axis at resting and the vertical line are considered to be the reference values for comparisons. Under induced boundary conditions of the pressures and active muscle contraction forces during micturition, for healthy (asymptomatic) pelvic floor, the displacements of the urethrovesical junction are measured to be VUVJ = 3.76 mm (inferiorly) and HUVJ = 3.44 mm (posteriorly). The angle Ur_(Mict)_ of the urethral axis with the vertical direction increased to 29.25°, and the difference of ΔUr = Ur_(Mict)_ − Ur_(Rest)_ = 7.25° is in agreement with the X-ray examination of 8° during micturition (Figure 6).

Figure 7 shows the progressive movement of the bladder neck with increased tissue impairment. On Valsalva manoeuvre with an IAP of 40 cm of H_2_0, the bladder neck mobility is markedly large (16.22 mm inferiorly and 6.54 posteriorly). The angle *α* is measured to be 138° (Figure 8(b)). The results show that the movement of the UVJ is strongly increased after 50% weakness of all the supporting tissues (ligaments and fasciae). However, it is interesting to observe that the effect of the endopelvic fascia weakness alone is sufficient to cause large dislocations of the organs compared to the ligaments together (Figure 7). This represents the hypermobility of the organs and commonly initiates situations such as incontinence and prolapse.

### 3.2. Q-Tip Test for SUI

Clinically, the change in the urethral axis through the diagnostic Q-tip test is commonly considered as a measure to predict the type of SUI [68].  Figure 8(a) shows a phenomenal effect of tissue impairment on the change of the urethral axis, Ur. An isolated impairment of the endopelvic fascia resulted in a more evident increase of the urethrovesical junction mobility than weakened ligaments together including the pubourethral ligament which supports the mid-urethra. After 40% impairment of the fascial support, the urethral axis (Ur_(Mict)_) increased dramatically beyond 30° with the vertical line which is normally considered to be a basic reference to predict the SUI due to urethral and vesical hypermobility.

### 3.3. Levator Plate Angulation

**Figure 9:**
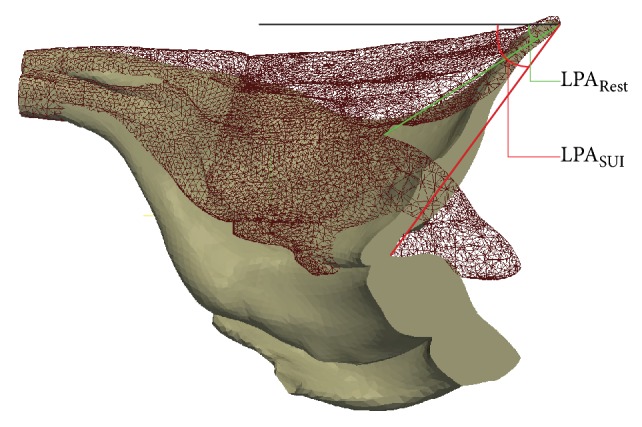
FE simulation results showing the sagittal section of increased levator plate angulation (LPA, the angle between the horizontal line and the line formed by two points between levator plate insertion point to the coccyx bone and anorectal junction) from rest (wireframe) to Valsalva manoeuvre (solid) for SUI female. ΔLPA = LPA_SUI_ − LPA_Rest_ = 22.5° is obtained between the rest (green) and during Valsalva manoeuvre (red).

To measure the levator plate angle (LPA), a best fit line is placed for the levator plate at the initial take-off portion of the iliococcygeus from the coccyx (Figure 2). Between this line and a horizontal reference line, the LPA (subtended by the green line and the horizontal line in Figure 9) at rest is measured to be 31.5°. In the female pelvic floor with healthy support, the measured levator plate angle is 42°. During Valsalva manoeuvre, women with simulated SUI showed statistically greater LPA compared to healthy tissue support (42° versus 54°) directed more caudally (Figure 9).

### 3.4. Discussions

Pelvic floor dysfunctions (PFD) have been commonly expected to be due to weakness, overstretching, or denervation injuries of the ligamentous structures, fasciae, organs themselves, and the pelvic musculature [1, 8, 11, 13]. Histological, experimental, and computational studies have been performed on samples of the organs, ligaments, and muscles in order to characterize their biomechanical response and to predict the development of the PDFs. However* the influence of altered fascial properties due to proportional reduction of elastin and collagen* on the mobility of the pelvic organs can hardly be visualized via radiological techniques; hence they have never been investigated. The numerical studies were performed on the presented model of the endopelvic fascia, differentiated with respect to varying elastin and collagen content, which supports the hammock hypothesis. The results in this study showed that it is the endopelvic fascia which covers most part of the pelvic diaphragm which bears the induced IAP and whose structural integrity is mainly responsible for the restriction of relative motion of the pelvic organs.

For normal micturition with intact support system, ΔUr = 7.25° is in agreement with the MRI examinations (Figures 5 and 6). The movement of the UVJ is 5.1 mm for the healthy support system in our study which is in good agreement with the MRI examination (5 mm) of a nulliparous (asymptomatic or healthy or continent) female without tissue impairment [22].  Table 4 illustrates the measurement of the UVJ and the angle alpha *α*. The measured values for the healthy female and for SUI simulations are within the ranges which are reported in the cited literature. The measured difference (22.5°) of the angle alpha between healthy and SUI Valsalva manoeuvre is similar to the references (Table 4). Also, since the computed pelvic model has been constructed from a 72-year-old female with no history of pelvic pathology, *α*_rest_ > 110° for an elderly female is reasonable.

Results of the FE simulations validate the positive Q-tip test. An urethral angle of Ur > 30° during exertional activities indicates a hypermobile urethrovesical junction. Weakness of the endopelvic fascia after 40% impairment is found to develop SUI (Figure 8(a)). Through representing a different physiopathological situation, a similar effect of the fascia impairment has been recently observed for the phenomenal development of the cystocele. Symptoms are dramatic after 40% impairment of the pubocervical fascia [74].

Petros emphasized the difference between the micturition in continent females and active opening in incontinent females; the latter is induced by a weak support system during increased IAP. Bush et al. [39] found that the normal detrusor pressure alone is not sufficient to open the urethra in contrast to the conventional theory, which assumes that the female urethra opens uniquely because of the detrusor pressure. The numerical results presented in this paper support the proposition of the Integral Theory that, together with an active muscle mechanisms, the increase of the detrusor pressure by two orders of magnitude is required to achieve the urethral funneling and urethrovesical hypermobility (Figure 5). However, the mechanical stiffness around the bladder neck has not been reduced in our study, since increased bladder pressure alone resulted in urethral funneling. In addition, weakness of the internal urethral orifice involves a different form of SUI arising from the defect of the internal urethral sphincter, which is different due to damaged ligaments, muscles, and fasciae. Also, the proximal 2/3rd of the urethral length and the lower portion of the urinary bladder is lined by transitional epithelium cells while the distal 1/3rd of the urethra is lined by stratified squamous epithelium cells [75]. Relatively softer transitional epithelium cells might open the urethral sphincter and move the proximal urethra more than the distal end.

Angulation of the levator plate in craniocaudal direction is obvious during pelvic manoeuvres due to increased IAP and active muscle mechanisms. MRI examinations of patients with genuine SUI measured an increased angle ΔLPA = 24.7° from rest (13.2° ± 11.9°) to straining (−11.5° ± 15.5°) [78], where negative values represent a bladder neck inferior to the pubococcygeal line and positive values represent a bladder neck above the pubococcygeal line. During simulated Valsalva manoeuvre in this study, a 22.5° change in the LPA has been calculated (Figure 9) which is in total agreement with the MRI examination.

### 3.5. Future Work

The presented model showed promising results to predict the movement of the pelvic organs and validated the normal micturition and active opening during SUI. However some anatomical and functional differences in the numerical results still exist and are discussed in detail.

#### 3.5.1. Performance of the Mucous Anorectal Cavity

In the simulation model, all pelvic organs except the uterus are created as hollow organ with anatomical thickness (Figure 2). These organ cavity walls are covered with visceral fluids or mucus which acts as lubricant and protects pelvic structures against infection. One of the largest cavities, the anorectal cavity in this study, is completely emptied without any faeces after defecation. During Valsalva manoeuvre, the mucous overlaid cavity might change numerical results quantitatively once the walls of the cavity are in contact under high deformation. All contacts can be simulated without friction and calculation of self-contact should not pose a problem if the rectum is empty. The finite element analysis performed in this study is focused on the positions of the urethra and the bladder for healthy and stress urinary incontinent patients. Therefore, in this study such extreme deformation of the anorectal walls is not considered. However, the presented model can be extended in studies of other problems such as rectal incontinence or rectocele where such movement is observed.

#### 3.5.2. Tail Bone Flexion Causing Further Descent of LP

Petros [36] hypothesized that the backward pull of the levator plate from the muscle fibres during pelvic manoeuvres widens the levator hiatus and causes organ descent. In numerical studies, the ligaments, the levator plate, and the coccygeus muscles connected to the coccyx are completely constrained in all directions. However, studies showed that the flexion angle between the coccyx and the sacrum body changes about 9.3°–20° [79, 80]. This flexion of the coccyx during increased IAP might cause the posterior shifting of the levator plate which increases the levator hiatus length and the levator plate angulation. Since the PFDs are mainly based on the positions of the organs and muscles, the coccygeal flexion should also be investigated in clinical examinations and adopted in the numerical analysis.

#### 3.5.3. Tissue Orthotropy

Almost all biological soft tissues are composed of elastin and collagen fibres embedded in the ground substance with varying amount of adipose tissue and smooth muscle depending on the anatomical location and function. A pronounced anisotropy is associated with such tissues and is the main difficulty in the biomechanical modelling. However, assuming that the pelvic structures are dominantly subjected to uniaxial tension along the fibre direction during pelvic movement, an isotropic formulation adopted in this study is appropriate.

One of the reasons to adopt the isotropic hyperelastic tissue behaviour in this study is the lack of sufficient experimental data for each and every pelvic tissue. Histological studies and multiaxial deformation tests have not been performed for all pelvic tissues; only few specimens extracted either from humans or from animals are tested in such way [56–60]. We have started to identify orthotropic data with own tests of porcine intestine and could relate the local behaviour with the specific function of the different sections of the organ. However, we have not found published data on the orthotropic behaviour of all structures in the human pelvic floor.

The required function in the study is the load carrying and not the function of the organs themselves. Therefore, a curve fit of tension tests in the direction of the dominating load direction with isotropic material laws can provide realistic representations of the load transfer in the pelvic floor. On this route we could base our analysis on a careful selection of recently published data of all organs in the model. Therefore, our analysis can be compared with studies of other research groups which used the same isotropic material models because all our improvements can be related to our most detailed representation of the geometry and the novel inclusion of the fasciae.

We have also implemented the orthotropic hyperelastic Holzapfel et al. model [40] in* Code_Aster* which represents the effect of fibre orientation in the tissues and made some developments to overcome unphysical behaviour which may occur with this material model [81]. Recently we could show that the orthotropic compressible behaviour of two mesh implants for hernia repair could be represented by the polyconvex Itskov material model [82]. Further testing and implementation in the FEM code are planned. Since biaxial material data on human or animal pelvic tissues are not completely tested yet, our pelvic floor model could be adapted to orthotropic tissue behaviour as soon as data becomes available.

## 4. Conclusions

A three-dimensional computer model presented in this numerical study shows its capability to better understand the dynamics of the female pelvic floor and the phenomena of stress urinary incontinence. Numerical simulations showed that the weakness of the heterogeneous network of the endopelvic fasciae genuinely causes the abnormal dislocation of the pelvic organs. The phenomena of the SUI are associated with the urethral hypermobility, downward and clockwise urinary bladder rotation, rotation of the urethral axis towards the horizontal position, and greater levator plate angulation during physiological Valsalva/straining manoeuvres which are observed in incontinent female patients. Thus, the model could be employed to predict the behaviour of the female pelvic floor, to plan the surgical treatment of the PFDs, and to minimize the postoperative complications after implantation of prosthetic meshes during minimally invasive surgeries.

## Figures and Tables

**Table 1:** Abbreviations used in the figures.

Abbr.	Full term
ANOC	Anococcygeal raphe
B	Bladder
CCM	Coccygeus muscle
CL	Cardinal ligament
Cx	Coccyx
EAS	External anal sphincter
ICM	Iliococcygeus muscle
Obt	Obturator internus muscle
Pb	Perineal body
PCM	Pubococcygeus muscle
Pm	Perineal membrane
PRM	Puborectalis muscle
Ps	Pubic symphysis
PUL	Pubourethral ligament
R	Rectum
U	Urethra
Umb	Umbilical ligament
USL	Uterosacral ligament
Ut	Uterus
V	Vagina

**Table 2:** Biomechanical properties of endopelvic fascia constituents.

Tissue component	*C*_10_ (MPa)	*C*_01_ (MPa)	*C*_20_ (MPa)	References
Collagen-elastin fascia	-	0.64785	-	[47]
Adipose (fat) tissue	0.000835	-	0.0128	[50]
Smooth muscle	0.0035	-	0.0155	[39]

**Table 3:** Biomechanical properties of pelvic structures obtained from the curve-fit of the experimented stretch-stretch curves adopted from cited literature. Polynomial equation (16) is used to derive parameters in our study.

Structure	*C*_10_ (MPa)	*C*_01_ (MPa)	*C*_20_ (MPa)	References
Vesica, urethra	0.0835	-	0.092	[61]
Uterus, vagina	0.4	-	3.2	[62]
Rectum	0.73	-	1.4	[62]
Cardinal ligament, perineal body	0.834	-	6.779	
Uterosacral ligament	1.6	-	8.0	[63]
Pubourethral ligament	0.68	-	5.0	[22, 63]
Collagen-rich endopelvic fascia, perineal membrane	-	0.64785	-	[47]
Elastin-rich endopelvic fascia	-	0.1619625	-	
Pelvic muscle	0.0625	-	-	[22]

In our study, the coefficients (*C*_*ij*_) for the uterosacral ligament are obtained by averaging the left and right ligaments. To our knowledge, mechanical characterization of the cardinal ligaments from human subjects has not been published. Therefore, we derived the parameters by comparing the relative differences with uterosacral ligaments in porcine specimen [64].

**Table 4:** Comparison of the measured urethrovesical junction $\left(UVJ=\sqrt{{\left(VUVJ\right)}^{2}+{\left(HUVJ\right)}^{2}}\right)$ movement and the angle between the midpubic line with the bladder neck (*α*). The difference of the angle alpha between healthy and SUI simulation is computed as Δ*α*_Valsalva_ = *α*_SUI_ − *α*_healthy_.

Reference	UVJ (mm)		Reference	*α* (°)			
	Healthy	SUI		*α*_rest_	*α*_healthy_	*α*_SUI_	Δ*α*_Valsalva_
Current simulation	5.1	17.5		110	115.5	138	22.5

Brandão et al. [22]	5.7	12.0	Brandão et al. [22]	91.8	105.7	124.3	18.6
Peschers et al. [69]	15 ± 10	-	Pregazzi et al. [70]	92.0 ± 6.0	100.0 ± 8	120.0 ± 8	20.0
Howard et al. [71]	12.4 ± 4.7	14.8 ± 6.4	Howard et al. [71]	103	-	-	-
Viereck et al. [72]	-	13.7 (2–30)	Peng et al. [18]	73	-	-	-
Brandt et al. [73]	-	VUVJ = 16.0 ± 6.8 HUVJ = 3.1 ± 1.0					
